# Acquired somatic variants in inherited myeloid malignancies

**DOI:** 10.1038/s41375-022-01515-2

**Published:** 2022-02-09

**Authors:** Hannah Armes, Ana Rio-Machin, Szilvia Krizsán, Csaba Bödör, Fadimana Kaya, Findlay Bewicke-Copley, Jenna Alnajar, Amanda Walne, Borbála Péterffy, Hemanth Tummala, Kevin Rouault-Pierre, Inderjeet Dokal, Tom Vulliamy, Jude Fitzgibbon

**Affiliations:** 1 Centre for Genomics and Computational Biology, Barts Cancer Institute, Queen Mary University of London, London, UK; 2 HCEMM-SE Lendulet Molecular Oncohematology Research Group, 1st Department of Pathology and Experimental Cancer Research, Semmelweis University, Budapest, Hungary; 3 Centre for Genomics and Child Health, Blizard Institute, Queen Mary University of London, London, UK; 4 Centre for Haemato-Oncology, Barts Cancer Institute, Queen Mary University of London, London, UK

**Keywords:** Acute myeloid leukaemia, Genetics research, Cancer genetics, Myelodysplastic syndrome

## To the Editor:

Inherited myeloid malignancies are considered a rare disease entity that exhibit significant heterogeneity in penetrance, age of onset and clinical presentation. When exploring disease evolution in this setting, determination of a causal germline variant represents one piece of the puzzle, yet the clinical heterogeneity we observe, even within families, suggests that germline carriers are also influenced by the acquisition of somatic mutations. Despite advances in the identification of germline variants, our understanding of the landscape of acquired mutations in familial myelodysplastic syndrome (MDS) and acute myeloid leukaemia (AML) remains incomplete.

We recently performed an analysis of the largest cohort of MDS/AML families to date, utilising next-generation sequencing technologies to profile the coding genome and identify inherited lesions in families with a clinical history suggestive of germline involvement [1]. By looking at previously defined disease-causing loci, we identified putative pathogenic germline variants in 57% of families across 16 genes.

In the current study, we focused our attention on acquired mutations within a cohort of 33 well-characterised MDS/AML families where ≥2 members were diagnosed with a haematological disorder, of which ≥1 case was specified as MDS/AML. DNA samples corresponding to 51 individuals from these families were available for the study (Supplementary Fig. S1). Of these, 16 were diagnosed with AML, 22 MDS, 1 thrombocytopenia (TCP), 7 bone marrow failure (BMF), 1 lymphoedema, 1 had abnormal lymphocyte subsets and 3 were asymptomatic carriers (Supplementary Table S1, Supplementary Fig. S1). Each individual carried a germline variant in 1 of 13 discrete loci known to predispose to familial MDS/AML (Supplementary Fig. S2). The age of onset of MDS/AML was highly variable (median, 30 years; range, 1–76 years) and dependent on the nature of the germline mutations. *RUNX1*, *CEBPA* and *GATA2* families exhibited early-onset MDS/AML, with a median age of 10, 18 and 21 years at presentation, respectively, compared with *TERT/TERC, SRP72* and *DDX41* families who had a longer latency, and a median age of 41.5, 51 and 56 years, respectively (Supplementary Fig. S3).

**Fig. 1:**
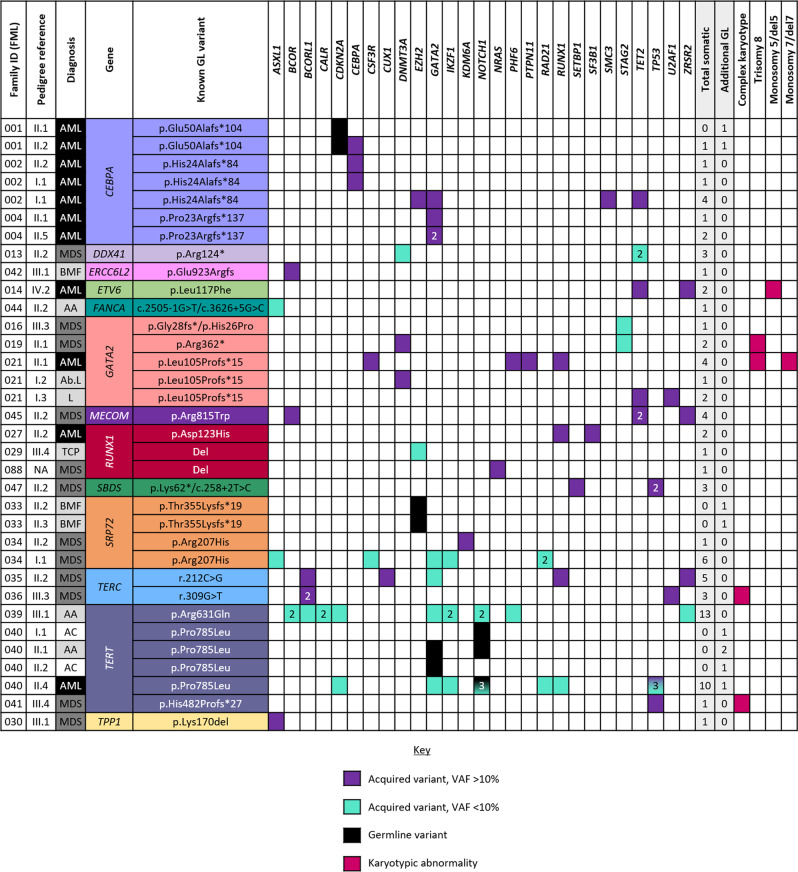
Aggregation of the acquired mutations identified in our series of patients. Only patients with one or more acquired or additional germline variant in our series are included. Purple represents mutations with a VAF > 10%; turquoise, VAF < 10%; black, additional germline variants and pink represents karyotypic abnormalities. Abbreviations: AA aplastic anaemia, Ab. L abnormal lymphocyte subsets, AC asymptomatic carrier, AML acute myeloid leukaemia, BMF bone marrow failure, GL germline, L lymphoedema, MDS myelodysplastic syndrome. For further details of somatic mutations in our series refer to Supplementary Table S3.

Acquired mutations were assessed using a commercial 54-gene TruSight Myeloid Sequencing Panel (Illumina; Supplementary Table S2), performed at a mean depth of 1,300 reads following the filtering criteria summarised in Supplementary Fig. S4 (data available in the European Nucleotide Archive; Accession Number: PRJEB49554). In total, 78 acquired mutations passed our filtering criteria, corresponding to 27 of the 54 genes analysed (Fig. 1; Supplementary Table S3), with 41 (53%) of these catalogued as somatic mutations in COSMIC and 59 (76%) absent in control populations. At least one acquired mutation was identified in 28/51 individuals including 10 AML and 12 MDS patients, and the median variant allele frequency (VAF) of the acquired mutations was 11.7% (range, 5.2–99.5%).

To better define the spectrum of acquired mutations in inherited myeloid disorders, we performed an integrative analysis of the acquired mutational signature in our cohort in addition to published families harbouring germline variants in the same 13 predisposing loci. We have included 395 MDS/AML patients from across 64 publications (Supplementary Fig. S5; Supplementary Table S4), and even accounting for differences in patient inclusion, sequencing methodology and analysis we observed a high concordance between our results and previously published families.

The integrated results, summarised in Fig. S6, demonstrate the heterogeneity of acquired mutations that exists in familial myeloid malignancies. Overall, this analysis confirmed previous observations of the striking frequency of second-hit *RUNX1, CEBPA* and *DDX41* mutations in patients harbouring these germline variants, recurrence of monosomy 7, trisomy 8, and *STAG2* and *ASXL1* mutations in *GATA2* families, and acquired *GATA2* mutations secondary to germline *CEBPA* variants. We also observed a striking prevalence of acquired *TP53* mutations in *SBDS* patients, with several individuals exhibiting multiple *TP53* mutations, and a high frequency (>80%) of *DDX41-*mutated cases presenting as cytogenetically normal (Supplementary Table S4). It is harder to apportion importance to many other acquired mutations as they arise infrequently and the size and number of patient cohorts remain low overall. That said, it is worth recognising the relevance of secondary variants in defining clinical manifestations in inherited malignancies, for instance, *ETV6*-mutated AML exhibits a distinct somatic profile to acute lymphoblastic leukaemia (ALL) even when arising from the same germline variant, suggesting that cooperating mutations can impact whether a neoplasm develops in the lymphoid or myeloid lineage [2].

Our study is the first to report the nature of acquired variants in *SRP72* families (FML033: p.Thr355Lysfs*19; FML034: p.Arg207His). While both families exhibited similar clinical manifestations, presenting with BMF or TCP in childhood/early adulthood, with some individuals developing MDS in adulthood [3], we observed inter- and intra-familial heterogeneity in their mutational profiling. Two siblings from FML033 (II.2, II.3) with congenital nerve deafness and presenting with BMF at 12 and 11 years, respectively, harboured an *EZH2* germline variant (p.Met191Val, uncertain significance, gnomAD frequency: 8.8 × 10^−6^) but no cooperating acquired mutations. In FML034, the index case (II.2) was diagnosed with refractory anaemia with excess blasts (MDS-RAEB) at 51 years and harboured a single *KDM6A* variant (p.Ser1192Pro, absent in gnomAD; VAF = 50%, predicted pathogenic), while her mother (I.1), who also developed MDS in her 70’s, harboured six low-VAF secondary variants (*ASXL1*, *CSF3R*, *GATA2*, *IKZF1* and two variants in *RAD21*; all with VAF < 10%) (Supplementary Table S3).

**Fig. 2:**
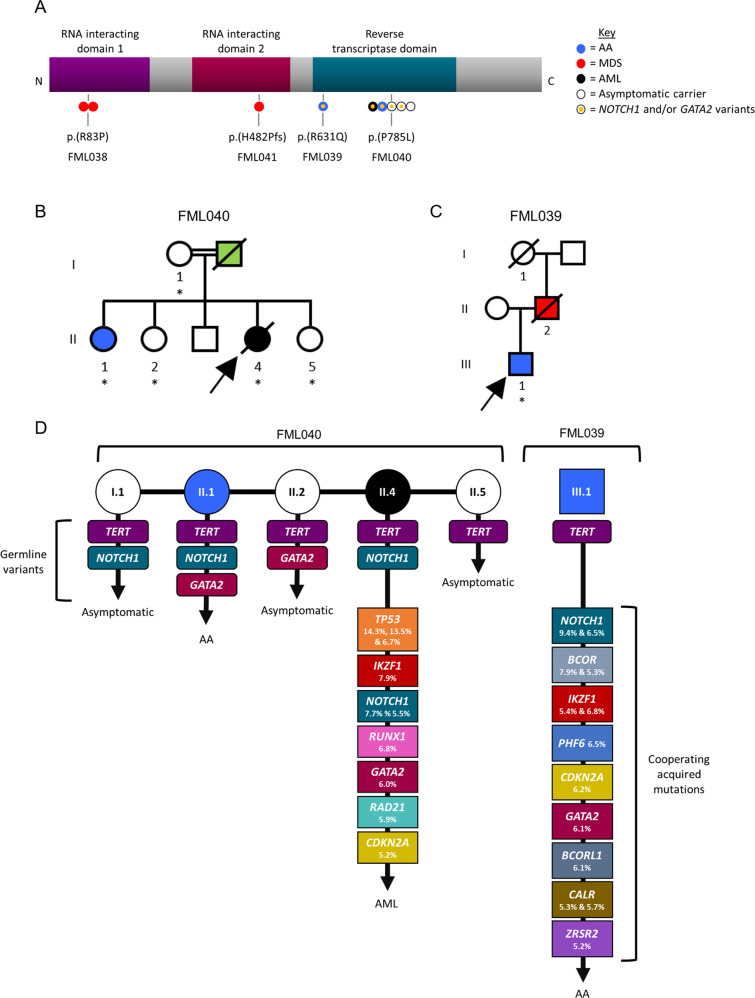
Analysis of *TERT* families. **A** Schematic of the TERT protein. Location of germline variants of 9 individuals in our series are depicted in relation to the RNA-interacting and catalytic domain(s). Circles represent individual cases and are colour coded by disease phenotype. **B**, **C** Schematic representation of *TERT* families, FML040 and FML039. Black represents AML; red, MDS; blue, bone marrow failure and green, other cancer. Samples included for targeted sequencing are indicated with an asterisk. In FML040, individuals I.1, II.2 and II.5 are asymptomatic carriers of the *TERT* germline variant. In FML039, individual I.1 is also an asymptomatic carrier. **D** Variants identified in *TERT* families, FML040 and FML039. Germline variant pattern (*TERT, GATA2, NOTCH1*), cooperating acquired mutations with corresponding VAF and patient phenotype (AML, AA or asymptomatic) are indicated.

Importantly, our study also identifies a novel mutational signature in *TERT* families for the first time. Four families with germline *TERT* variants were included in our series (FML038, FML039, FML040 and FML041), corresponding to six patients with aplastic anaemia (AA) (*N* = 2), MDS (*N* = 3) and AML (*N* = 1), and three asymptomatic carriers. Fig. 2A shows a schematic of the location of the four *TERT* germline variants: FML038 (p.Arg83Pro) and FML041 (p.His482Profs*27) harbour heterozygous missense and truncating variants, respectively, within the N-terminal RNA-interacting domains, while families FML039 (p.Arg631Gln) and FML040 (p.Pro785Leu) (Fig. 2B, C) retained heterozygous missense *TERT* variants located within the C-terminal catalytic reverse transcriptase domain (RTD) responsible for maintaining telomere ends. Carriers of the RTD *TERT* variants in FML039 and FML040 were previously found to exhibit short telomeres and abolished/reduced telomerase activity [4].

We observed a complex pattern of inheritance in FML040 suggesting that the clinical presentation in this family may arise from germline variants at three discrete loci, including *TERT, NOTCH1* and *GATA2* (Fig. 2D; Supplementary Fig. S7). *TERT* and *NOTCH1 (*p.Pro2128Leu, uncertain significance, gnomAD frequency: 7 × 10^−5^) variants were inherited from the asymptomatic mother in two offspring, including the index case who developed secondary AML (II.4), and her sister with a diagnosis of AA (II.1). Sibling II.1 also inherited a germline *GATA2* variant (p.Pro14Ser; predicted damaging in SIFT and MutationTaster, gnomAD frequency: 4.8 × 10^−5^) that was also present in her asymptomatic sister (II.2) which we presume was inherited from her father (I.II). The genetic pattern is further complicated by the existence of acquired mutations in *NOTCH1* (VAF = 5.5% p.Glu1636Lys, gnomAD frequency: 1.29 × 10^−5^, COSM308616; and VAF = 7.7% p.Ala1634Asp, absent in gnomAD, COSM308589) and *GATA2* (VAF = 6.0% p.Phe400Leu, absent in gnomAD and COSMIC) at low VAF in II.4. Therefore, in our current model we propose that the onset of overt symptoms may be conditional on the presence of both *NOTCH1* and *GATA2* variants, since the mother (I.1) and sibling (II.2) remain symptom-free and harbour a single *NOTCH1* or *GATA2* variant, respectively.

In support of this model, we found that the index case in family FML039 (III.1), with AA and harbouring a germline *TERT* variant in the RTD, also retained two acquired *NOTCH1* mutations (VAF = 6.5% p.Pro2551fs, absent in gnomAD, COSM6918461; and VAF = 9.4% p.Ser2499delinsSerPro, absent in gnomAD and COSMIC) and a *GATA2* mutation (VAF = 6.1% p.Glu398Lys, absent in gnomAD, COSM7119678). It is plausible that these low VAF mutations represent a subclonal population of cells that may serve as a reservoir population from which transformed cells later emerge. The combination of *NOTCH1* and *GATA2* together may act as a risk factor for development of myeloid disease when in conjunction with the germline *TERT* variant. Though the precise molecular mechanism for the functional interaction between *TERT, NOTCH1* and *GATA2* is open to speculation, telomerase has previously been shown to impact *NOTCH1* signalling [5] while *NOTCH1* directly regulates the expression of *GATA2* during endothelial-to-haematopoietic transition during development [6]. We also noted co-occurrence of somatic *IKZF1* and *CDKN2A* mutations with *NOTCH1* in the two index cases of the *TERT* families (FML039, FML040). This may not be surprising, since *IKZF1* appears to cooperate with NOTCH1 pathway activation to maintain homoeostasis of monocytic/dendritic progenitors [7] and T-ALL-activating Notch1 mutations in mice frequently coincide with loss-of-function mutations in *Ikzf1* [8–11]. This suggests there is value in reassessing other *TERT* families, particularly those with a germline RTD variant, to determine whether additional *NOTCH1* and *GATA2* mutations are a wider feature of these presentations or indeed, if other related mutations can also be detected in these families.

Altogether, we provide a comprehensive analysis of the somatic mutational landscape across a range of BMF and MDS/AML families, integrating the somatic profiling of 446 individuals from this study and previously published families with inherited variants in 13 gene loci. Our analysis demonstrates the importance of consortium efforts, championed by the RUNX1 foundation and Brown and colleagues [12], which enable trends of mutational acquisition to be identified in large cohorts of familial patients. Our dataset has provided insights into the complex interplay between genetic lesions that may contribute to disease development, particularly in the case of *TERT*-mutated families, suggesting that disease progression may be shaped by a particular combination of germline and somatic co-occurring variants. Above all, there is an unmet need to include familial loci for analysis in routine diagnostic panels, not only to document instances of inherited disease, but also to provide a resource to investigate acquired mutations and gain a greater understanding of the pathogenesis of both familial and sporadic disease.

## Supplementary information

Supplementary Material

Supplementary Table S2

Supplementary Table S3

Supplementary Table S4
