# Supplementary material for: Acquired somatic variants in inherited myeloid malignancies
Source: Leukemia. 2022 Feb 9;36(5):1377–81. doi: 10.1038/s41375-022-01515-2 (PMC9061295; doi:10.1038/s41375-022-01515-2)
Supplement: Supplementary file 1 — Supplementary Material [file 41375_2022_1515_MOESM1_ESM.pdf]

Acquired somatic variants in inherited myeloid malignancies.

Supplementary material

Supplementary Table S1. Clinical and genetic characteristics of our series of patients.

Supplementary Table S2. Details of the targeted sequencing panel.

Supplementary Table S3. Variants identified in our series of patients through the targeted sequencing of 54 myeloid genes.

Supplementary Table S4. Clinical and genetic characteristics of the literature cohort of patients.

Supplementary Figure S1. Schematic representation of 33 families included in our series.

Supplementary Figure S2. Percentages of our series with causal germline variants in the 13 loci.

Supplementary Figure S3. Age of MDS/AML onset in our series of patients.

Supplementary Figure S4. Schematic of the filtering criteria.

Supplementary Figure S5. Percentages of the literature cohort of MDS/AML patients with causal germline variants in the 13 loci.

Supplementary Figure S6. Integrative analysis to show acquired mutations in ours and published MDS/AML patients.

Supplementary Figure S7. Validation of germline variants in *TERT* families.

Supplementary References.

Known germline variant	Pedigree	Variant detail	ID	Sex	Age	Haematological malignancy	Pre-malignant disease and other manifestations
CEBPA	FML001	c.147_165del:p.Glu50Alafs*104	II.2	F	15	AML-M1	–
			II.1	F	1	AML-M1	–
	FML002	c.68dupC:p.His24Alafs*84	II.2	M	5	AML-M1	–
			I.1	F	37	AML-M1 (first relapse)	–
			I.1	F	40	AML-M1 (second relapse)	–
	FML004	c.68delC:p.Pro23Argfs*137	II.1	M	30	AML-M2	–
			II.5	F	18	AML-M2	–
DDX41	FML008	c.155dupA:p.Arg53Alafs*16	II.3	F	54	MDS	–
			III.1	F	49	MDS-RAEB	–
	FML010	c.1586_1587delCA:p.Thr529Argfs*12	II.2	F	58	AML	Pancytopenia
	FML011	c.3G>A:p.Met1? and c.-44G>A	II.1	F	41	MDS-RAEB	–
	FML012	c.3G>A:p.Met1?	II.2	M	68	MDS (RC)	–
	FML013	c.370C>T:p.Arg124*	II.2	M	68	MDS-RAEB	–
ERCC6L2	FML042	c.2767delG:p.Glu923Argfs*8	III.1	F	9	–	Hypocellular BM
ETV6	FML014	c.349C>T:p.Leu117Phe	IV.2	F	20	AML	–
FANCA	FML044	c.2505-1G>T/c.3626+5G>C	II.2	F	38	–	AA, carcinoma of the tongue
GATA2	FML016	c.83_83delG:p.Gly28fs* and c.77A>C:p.His26Pro	III.3	F	30	MDS	Leukopenia, TCP
	FML017	c.1187G>A:p.Arg396Gln	II.2	M	14	AML-M2	–
			II.1	M	16	MDS	–
	FML019	c.1084C>T:p.Arg362*	II.1	F	36	Hypoplastic MDS	Deafness
	FML020	c.1061C>T:p.Thr354Met	III.1	M	–	MDS	–
			II.2	F	26	MDS (RC)	–
	FML021	c.313_314insCC:p.Leu105Profs*15	I.2	M	–	–	Abnormal lymphocyte subsets
			I.3	F	44	–	Lymphoedema
			II.1	F	12	MDS to AML	Lymphoedema
MECOM	FML045	c.2443C>T:p.Arg815Trp	II.2	M	–	MDS	AA, radio-ulnar synostosis
RUNX1	FML025	c.958C>T:p.Arg320*	II.2	M	–	AML biphenotypic	–
	FML027	c.367G>C:p.Asp123His	II.2	F	29	AML	–
	FML029	Deletion	III.4	M	–	–	TCP
	FML089	Deletion	II.1	M	10	AML	TCP
	FML087	Deletion	–	–	–	AML	Risk of platelet disorder, global development delay
	FML088	Deletion	–	–	4	MDS	–
SBDS	FML047	c.258+2T>C and c.183_184delinsCT:p.Lys62*	II.2	F	23	MDS	Short stature, cleft palate, learning disabilities
SRP72	FML033	c.1064_1065del:p.Thr355Lysfs*19	I.1	F	45	MDS (RC)	Deafness
			II.2	M	12	–	BMF
			II.3	M	11	–	BMF
			II.1	F	12	–	BMF, deafness
	FML034	c.620G>A:p.Arg207His	II.2	F	51	MDS-RAEB	TCP
			I.1	F	76	MDS	–
TERC	FML035	r.212C>G	II.2	M	47	MDS	–
	FML036	r.309G>T	III.3	M	43	MDS	–

TERT	FML038	c.248G>C:p.Arg83Pro	III.1	F	26	MDS	AA
			II.2	M	53	MDS	–
	FML039	c.1892G>A:p.Arg631Gln	III.1	M	33	–	AA
	FML040	c.2354C>T:p.Pro785Leu	II.1	F	–	–	AA
			II.4	F	30	MDS to AML	–
	FML041	c.1445delA:p.His482Profs*27	III.4	M	40	MDS	Vasculitis
TPP1	FML030	c.508_510delAAG:p.Lys170del	III.1	F	–	MDS	–

Supplementary Table S1. Clinical and genetic characteristics of our series of patients. Abbreviations: AA, aplastic anaemia; AML, acute myeloid leukaemia; BMF, bone marrow failure; F, female; M, male; MDS, myelodysplastic syndrome; RAEB, refractory anaemia with excess blasts; RC, refractory cytopenia; RCMD, refractory cytopenia with multilineage dysplasia; TCP, thrombocytopenia. More information can be found in Supplementary Figure S1 and Supplementary Table S3. Variant nomenclature follows the recommendations of the Human Genome Variation Society (2016) (http://varnomen.hgvs.org/). The nomenclature of the germline *CEBPA* variants has been modified from our previous publications, accordingly (1, 2).

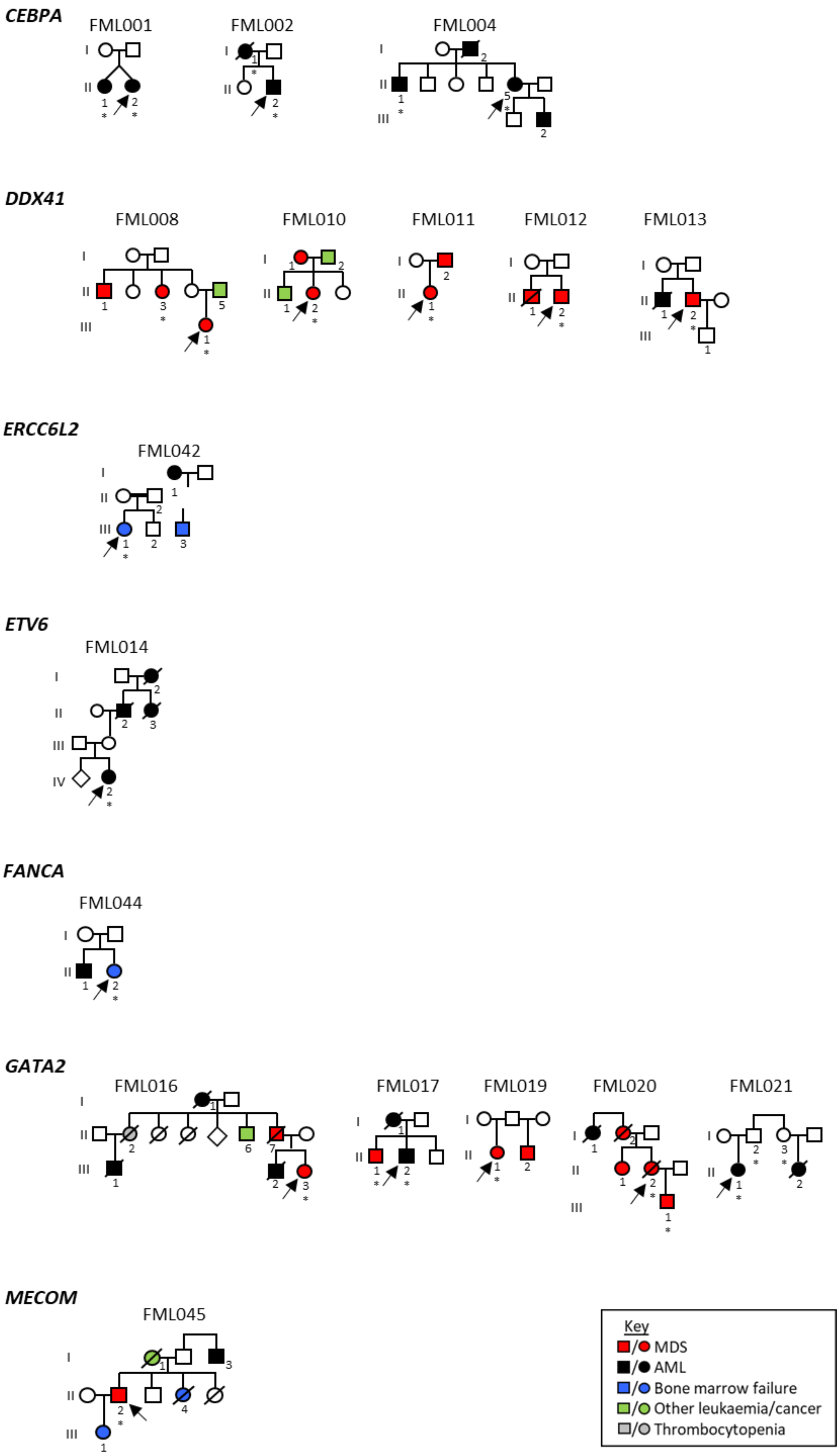
CEBPA
FML001
FML002
FML004
DDX41
FML008
FML010
FML011
FML012
FML013
ERCC6L2
FML042
ETV6
FML014
FANCA
FML044
GATA2
FML016
FML017
FML019
FML020
FML021
MECOM
FML045
Key
MDS
AML
Bone marrow failure
Other leukaemia/cancer
Thrombocytopenia

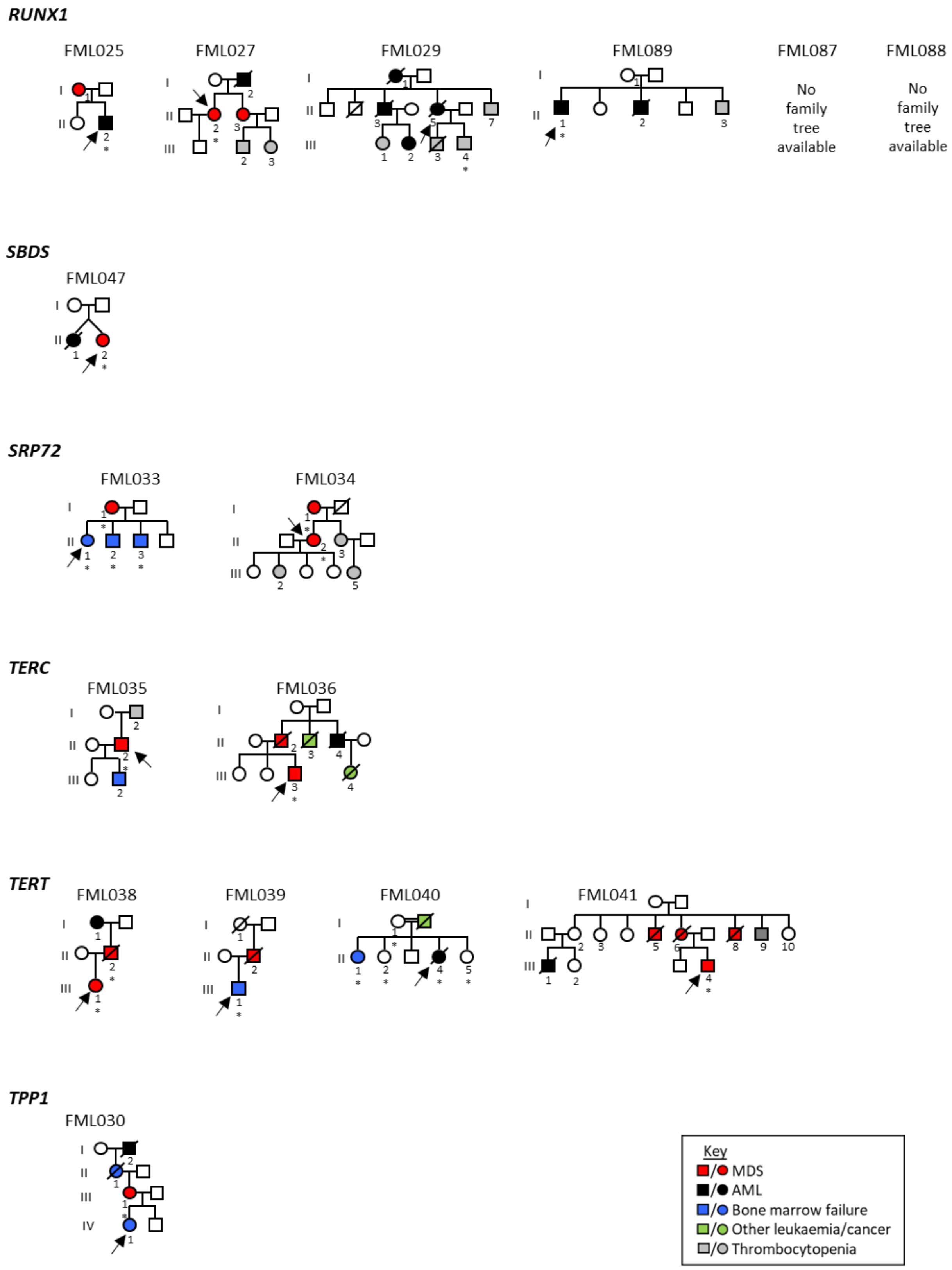

Supplementary Figure S1. Schematic representation of 33 families included in our series. Schematic representation of the 33 families included in this study, where a germline variant has been detected in a locus with a high/moderate level of evidence for gene-disease association. The index case of each family is indicated with an arrow. The samples that underwent targeted sequencing using the commercial 54-gene TruSight Myeloid Sequencing Panel are indicated with an asterisk.

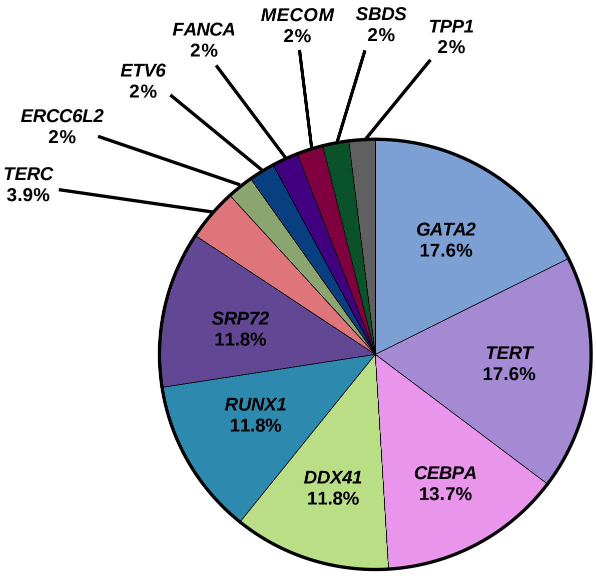

Supplementary Figure S2. Percentages of our series with causal germline variants in the 13 loci. Pie chart depicting the percentage of our series with causitive germline variants detected in one of the 13 loci (N=51).

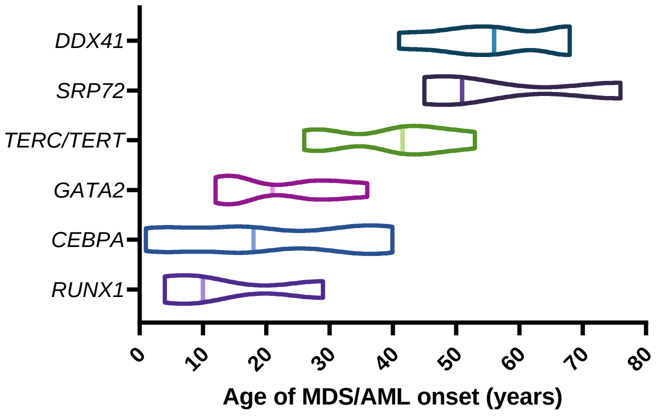

Supplementary Figure S3. Age of MDS/AML onset in our series of patients. Schematic of the age of MDS/AML onset in our series of patients separated by causal germline variant. Median values are shown.

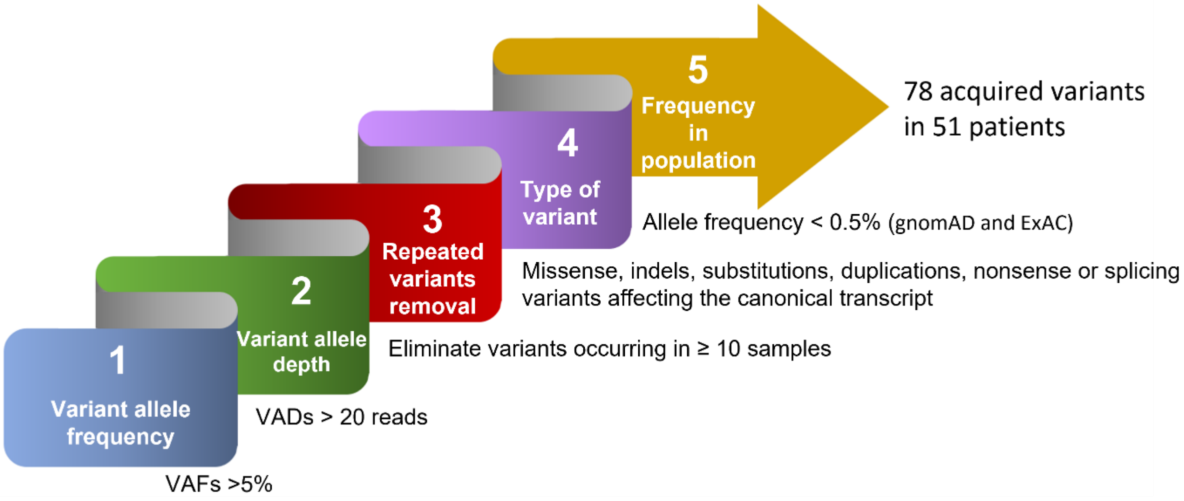

Supplementary Figure S4. Schematic of the filtering criteria. In brief, we removed all variants in the 1000 Genomes project (1000G) or NHLBI GO Exome Sequencing Project (ESP) with a frequency of >0.5%, and retained non-synonymous exonic or splicing calls with a variant allele frequency (VAF) greater than 5%. Variants that occurred in 10 or more samples or those with a sequencing depth less than 20 were removed. Mean sequencing depth was 1300 reads/locus. Targeted panel included the following 54 genes: *ABL1, ASXL1, ATRX, BCOR, BCORL1, BRAF, CALR, CBL, CBLB, CBLC, CDKN2A, CEBPA, CSF3R, CUX1, DNMT3A, ETV6/TEL, EZH2, FBXW7, FLT3, GATA1, GATA2, GNAS, HRAS, IDH1, IDH2, IKZF1, JAK2, JAK3, KDM6A, KIT, KRAS, MLL, MPL, MYD88, NOTCH1, NPM1, NRAS, PDGFRA, PHF6, PTEN, PTPN11, RAD21, RUNX1, SETBP1, SF3B1, SMC1A, SMC3, SRSF2, STAG2, TET2, TP53, U2AF1, WT1* and *ZRSR2*.

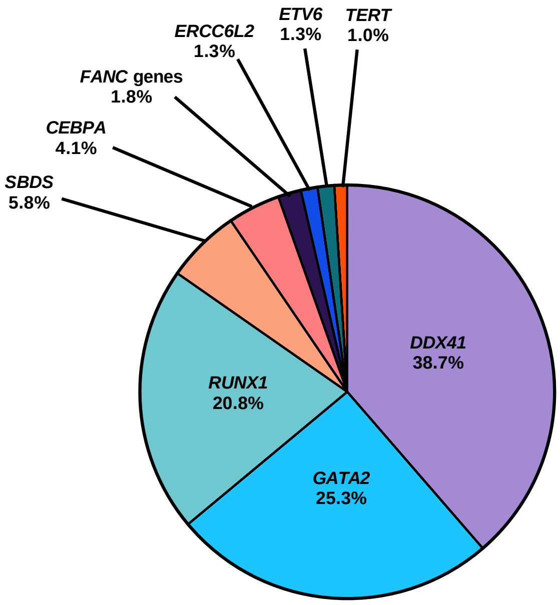

Supplementary Figure S5. Percentages of the literature cohort of MDS/AML patients with causal germline variants in the 13 loci. Pie chart depicting the percentage of our literature cohort with causative germline variants detected in one of the 13 loci (N=395). Inclusion criteria: diagnosis of MDS/AML, germline variant confirmed, sample included for analysis of acquired mutations, germline variant predicted to be damaging in ≥2 out of 4 tools for functional annotation (MutationTaster, Polyphen2, PROVEAN, SIFT).

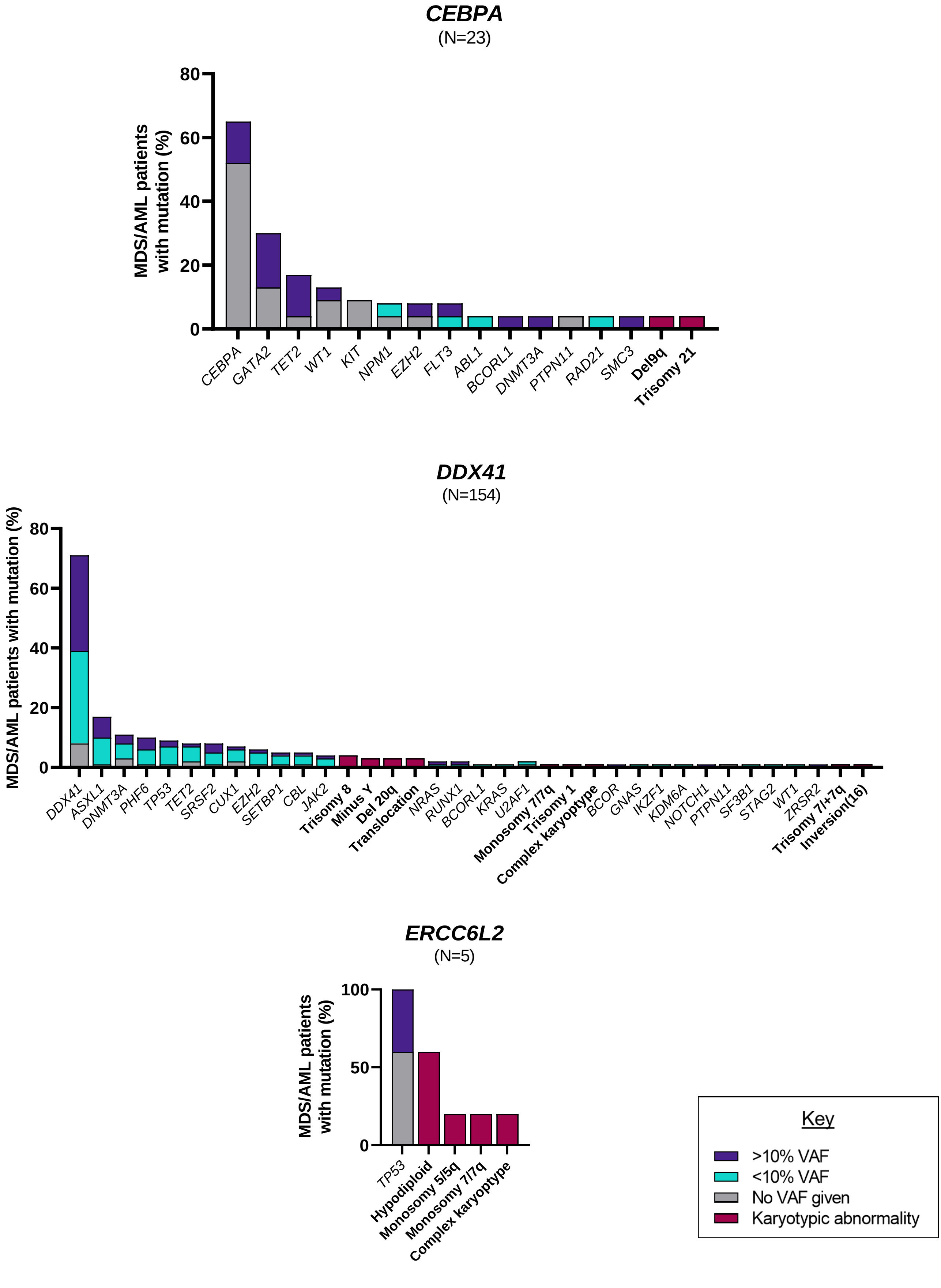
CEBPA
(N=23)
MDS/AML patients with mutation (%)
CEBPA
GATA2
TET2
WT1
KIT
NPM1
EZH2
FLT3
ABL1
BCORL1
DNMT3A
PTPN11
RAD21
SMC3
Del9q
Trisomy 21
DDX41
(N=154)
MDS/AML patients with mutation (%)
DDX41
ASXL1
DNMT3A
PHF6
TP53
TET2
SRSF2
CUX1
EZH2
SETBP1
CBL
JAK2
Trisomy 8
Minus Y
Del 20q
Translocation
NRAS
RUNX1
BCORL1
KRAS
U2AF1
Monosomy 7/7q
Trisomy 1
Complex karyoptype
BCOR
GNAS
IKZF1
KDM6A
NOTCH1
PTPN11
SF3B1
STAG2
WT1
ZRSR2
Trisomy 7/+7q
Inversion(16)
ERCC6L2
(N=5)
MDS/AML patients with mutation (%)
TP53
Hypodiploid
Monosomy 5/5q
Monosomy 7/7q
Complex karyoptype
Key
>10% VAF
<10% VAF
No VAF given
Karyotypic abnormality

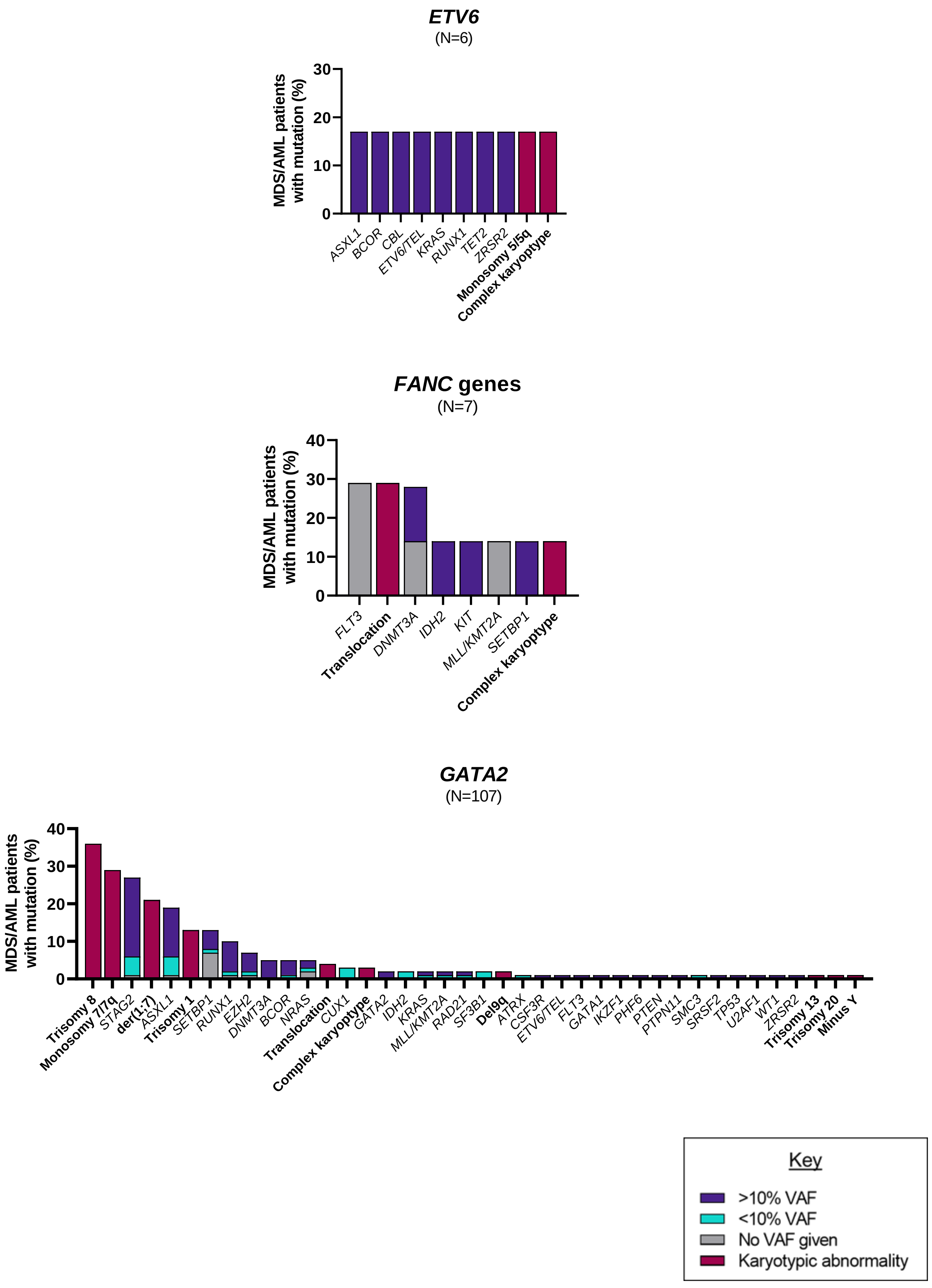
ETV6
(N=6)
MDS/AML patients with mutation (%)
0
10
20
30
ASXL1
BCOR
CBL
ETV6/TEL
KRAS
RUNX1
TET2
ZRSR2
Monosomy 5/5q
Complex karyoptype
FANC genes
(N=7)
MDS/AML patients with mutation (%)
0
10
20
30
40
FLT3
Translocation
DNMT3A
IDH2
KIT
MLL/KMT2A
SETBP1
Complex karyoptype
GATA2
(N=107)
MDS/AML patients with mutation (%)
0
10
20
30
40
Trisomy 8
Monosomy 7/7q
STAG2
der(1;7)
ASXL1
Trisomy 1
SETBP1
RUNX1
EZH2
DNMT3A
BCOR
NRAS
Translocation
CUX1
Complex karyoptype
GATA2
IDH2
KRAS
MLL/KMT2A
RAD21
SF3B1
Del9q
ATRX
CSF3R
ETV6/TEL
FLT3
GATA1
IKZF1
PHF6
PTEN
PTPN11
SMC3
SRSF2
TP53
U2AF1
WT1
ZRSR2
Trisomy 13
Trisomy 20
Minus Y
Key
>10% VAF
<10% VAF
No VAF given
Karyotypic abnormality

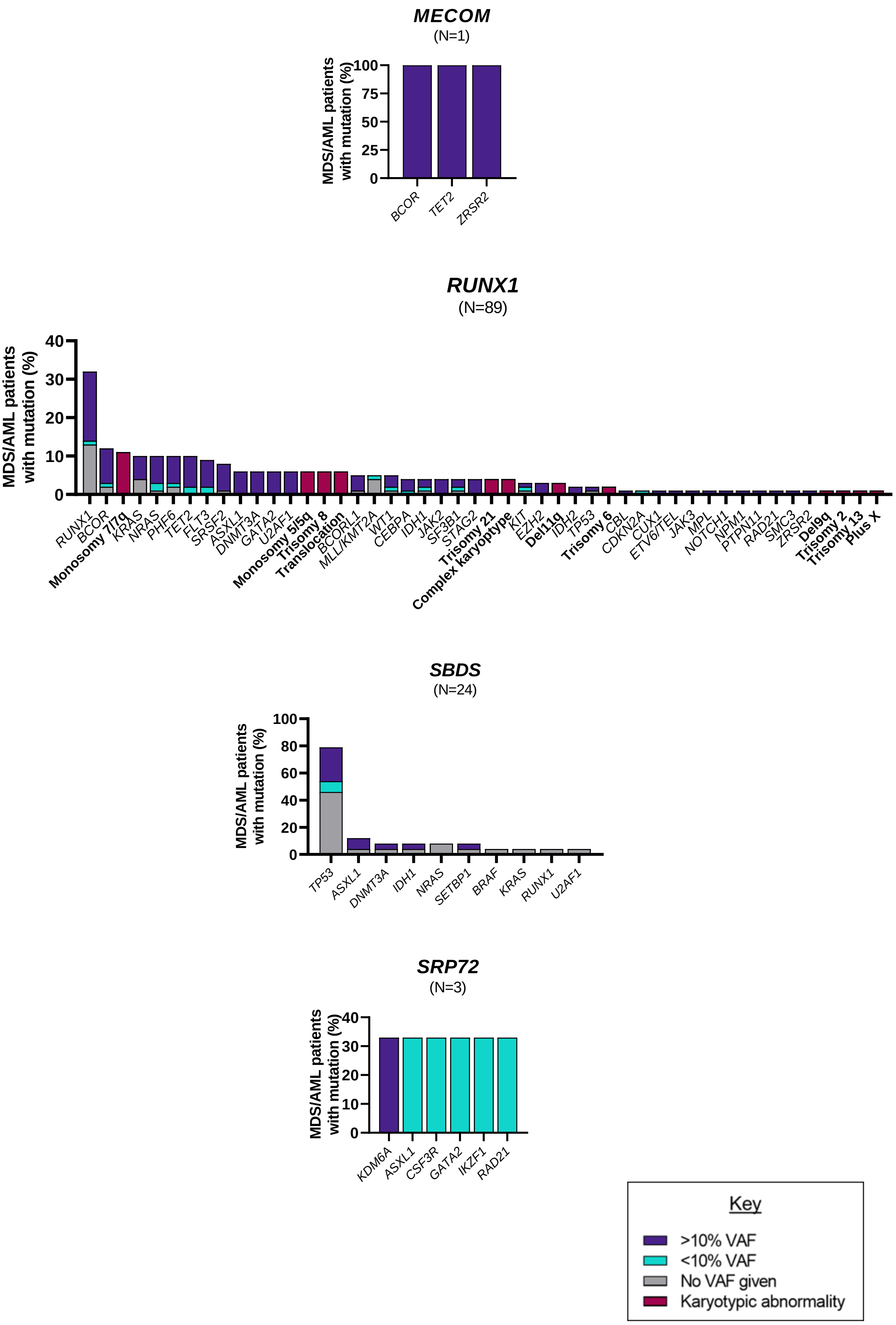
MECOM
(N=1)
MDS/AML patients with mutation (%)
0
25
50
75
100
BCOR
TET2
ZRSR2
RUNX1
(N=89)
MDS/AML patients with mutation (%)
0
10
20
30
40
RUNX1
BCOR
Monosomy 7/7q
KRAS
NRAS
PHF6
TET2
FLT3
SRSF2
ASXL1
DNMT3A
GATA2
U2AF1
Monosomy 5/5q
Trisomy 8
Translocation
BCORL1
MLL/KMT2A
WT1
CEBPA
IDH1
JAK2
SF3B1
STAG2
Trisomy 21
Complex karyoptype
KIT
EZH2
Del11q
IDH2
TP53
Trisomy 6
CBL
CDKN2A
CUX1
ETV6/TEL
JAK3
MPL
NOTCH1
NPM1
PTPN11
RAD21
SMC3
ZRSR2
Del9q
Trisomy 2
Trisomy 13
Plus X
SBDS
(N=24)
MDS/AML patients with mutation (%)
0
20
40
60
80
100
TP53
ASXL1
DNMT3A
IDH1
NRAS
SETBP1
BRAF
KRAS
RUNX1
U2AF1
SRP72
(N=3)
MDS/AML patients with mutation (%)
0
10
20
30
40
KDM6A
ASXL1
CSF3R
GATA2
IKZF1
RAD21
Key
>10% VAF
<10% VAF
No VAF given
Karyotypic abnormality

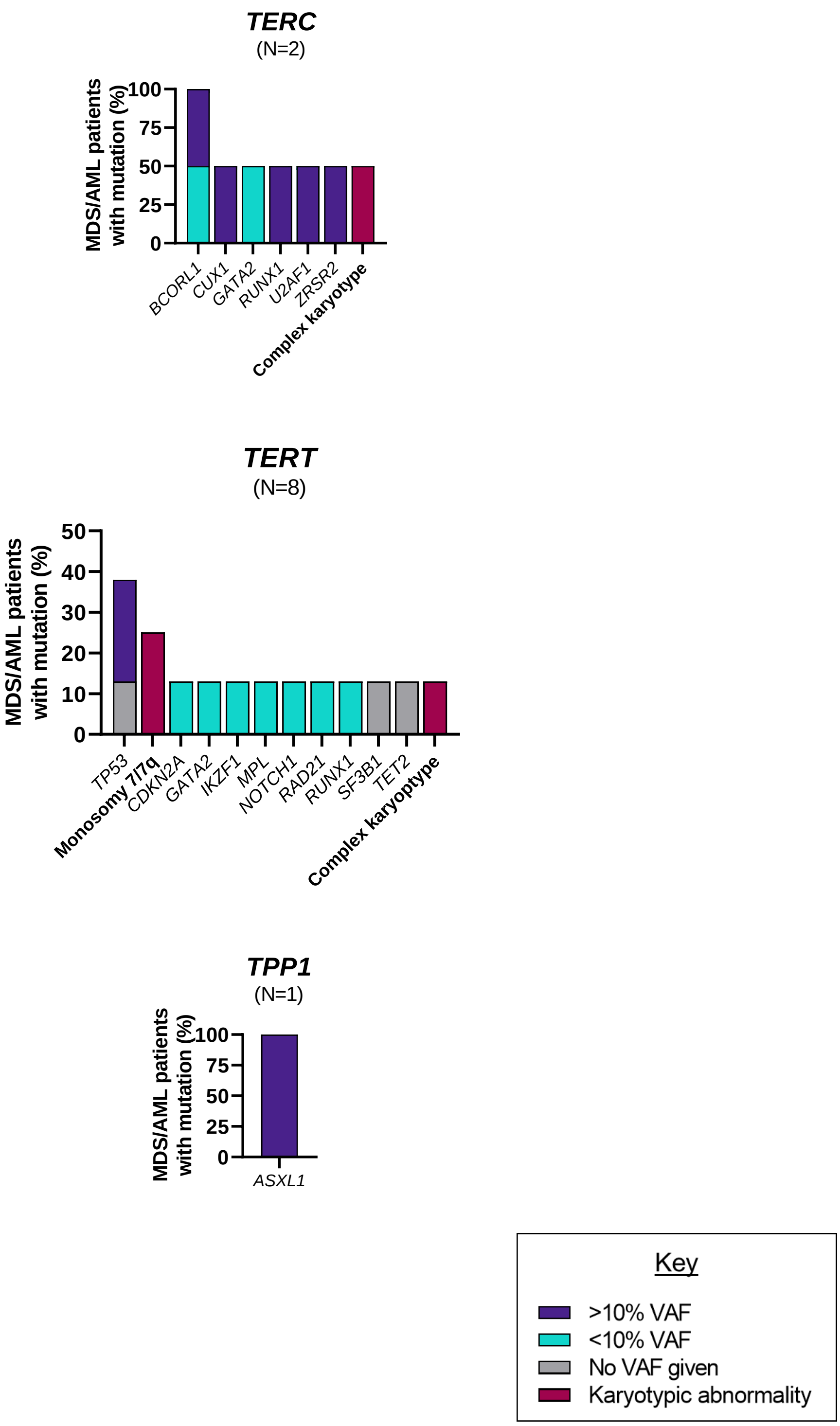

Supplementary Figure S6. Integrative analysis to show acquired mutations in ours and published MDS/AML patients. Integrative analysis of the acquired variant signature described in ours and published MDS/AML patients harbouring germline variants in 13 common predisposing loci. Data represents the percentage of MDS/AML patients with a mutation in each gene. Purple represents acquired variants with a VAF of >10%; turquoise, acquired variants with a VAF of <10%; grey, no VAF was provided; red, karyotypic abnormality. More information can be found in Supplementary Table S4.

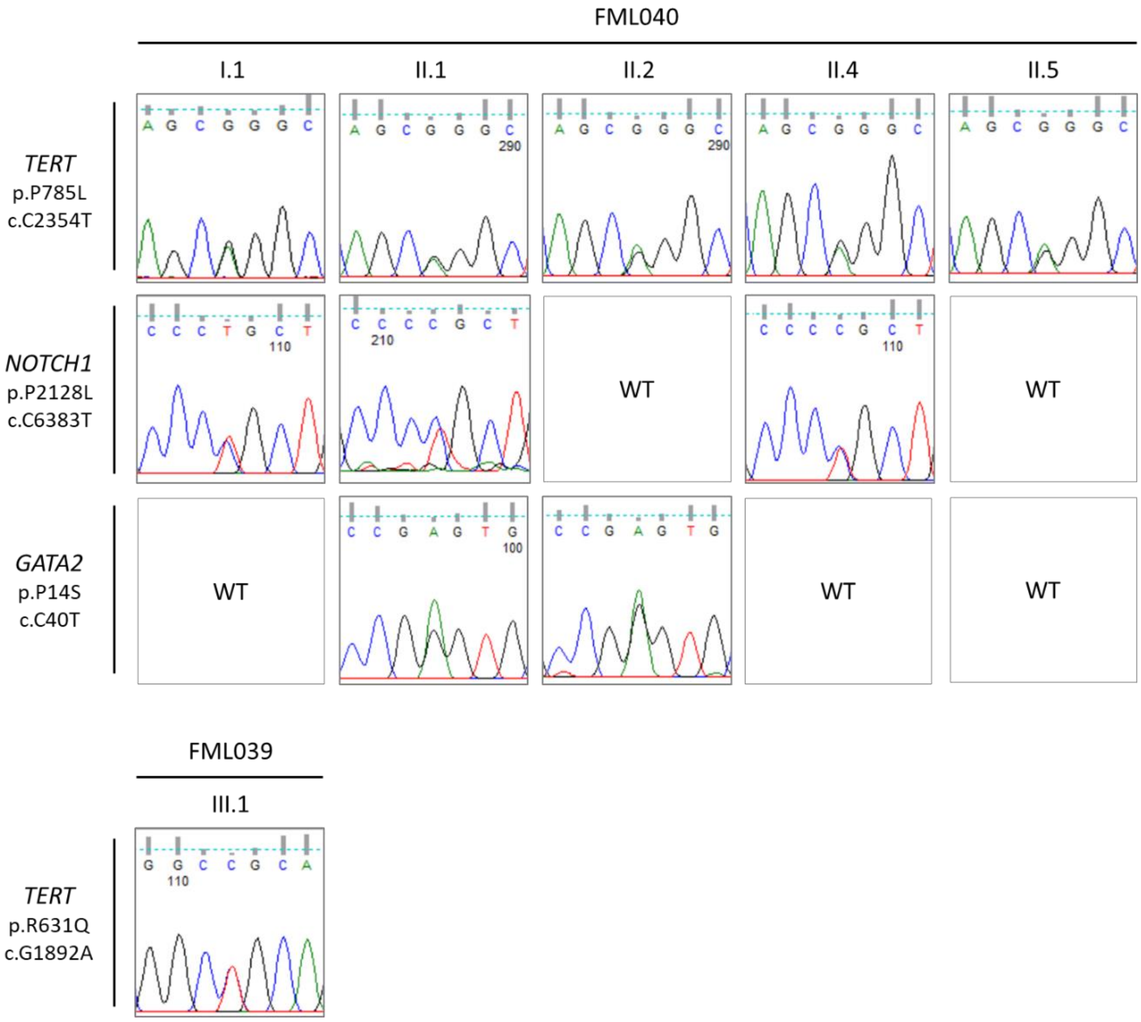

Supplementary Figure S7. Validation of germline variants in *TERT* families. Sanger sequencing traces of all the germline variants identified in *TERT* families, FML040 and FML039. WT, wild-type.

Supplementary references

1. Rio-Machin A, Vulliamy T, Hug N, et al. The complex genetic landscape of familial MDS and AML reveals pathogenic germline variants. Nat Commun. 2020;11(1):1044.

2. Tawana K, Wang J, Renneville A, et al. Disease evolution and outcomes in familial AML with germline CEBPA mutations. Blood. 2015;126(10):1214-1223.

3. Kim HS, Han E, Jang W, et al. Germline CEBPA mutations in Korean patients with acute myeloid leukemia. Leuk Res. 2019;76:84-86.

4. Yan B, Ng C, Moshi G, et al. Myelodysplastic features in a patient with germline CEBPA-mutant acute myeloid leukaemia. J Clin Pathol. 2016;69(7):652-654.

5. Zhang Y, Wang F, Chen X, et al. Companion gene mutations and their clinical significance in AML with double mutant CEBPA. Cancer Gene Ther. 2020;27(7-8):599-606.

6. Ram J, Flamm G, Balys M, et al. Index case of acute myeloid leukemia in a family harboring a novel CEBPA germ line mutation. Blood Adv. 2017;1(8):500-503.

7. Pabst T, Eyholzer M, Haefliger S, Schardt J, Mueller BU. Somatic CEBPA mutations are a frequent second event in families with germline CEBPA mutations and familial acute myeloid leukemia. J Clin Oncol. 2008;26(31):5088-5093.

8. Mendoza H, Chen PH, Pine AB, et al. A case of acute myeloid leukemia with unusual germline CEBPA mutation: lessons learned about mutation detection, location, and penetrance. Leuk Lymphoma. 2021;62(5):1251-1254.

9. Andrés-Zayas C, Suárez-González J, Rodríguez-Macías G, et al. Clinical utility of targeted next-generation sequencing for the diagnosis of myeloid neoplasms with germline predisposition. Mol Oncol. 2021;15(9):2273-2284.

10. Polprasert C, Schulze I, Sekeres MA, et al. Inherited and Somatic Defects in DDX41 in Myeloid Neoplasms. Cancer Cell. 2015;27(5):658-670.

11. Sébert M, Passet M, Raimbault A, et al. Germline DDX41 mutations define a significant entity within adult MDS/AML patients. Blood. 2019;134(17):1441-1444.

12. Qu S, Li B, Qin T, et al. Molecular and clinical features of myeloid neoplasms with somatic DDX41 mutations. Br J Haematol. 2021;192(6):1006-1010.

13. Abou Dalle I, Kantarjian H, Bannon SA, et al. Successful lenalidomide treatment in high risk myelodysplastic syndrome with germline DDX41 mutation. Am J Hematol. 2020;95(2):227-229.

14. Vairo FPE, Ferrer A, Cathcart-Rake E, et al. Novel germline missense DDX41 variant in a patient with an adult-onset myeloid neoplasm with excess blasts without dysplasia. Leuk Lymphoma. 2019;60(5):1337-1339.

15. Kobayashi S, Kobayashi A, Osawa Y, et al. Donor cell leukemia arising from preleukemic clones with a novel germline DDX41 mutation after allogenic hematopoietic stem cell transplantation. Leukemia. 2017;31(4):1020-1022.

16. Berger G, van den Berg E, Sikkema-Raddatz B, et al. Re-emergence of acute myeloid leukemia in donor cells following allogeneic transplantation in a family with a germline DDX41 mutation. Leukemia. 2017;31(2):520-522.

17. Li R, Sobreira N, Witmer PD, Pratz KW, Braunstein EM. Two novel germline DDX41 mutations in a family with inherited myelodysplasia/acute myeloid leukemia. Haematologica. 2016;101(6):e228-231.

18. Blombery P, Fox L, Ryland GL, et al. Utility of clinical comprehensive genomic characterization for diagnostic categorization in patients presenting with hypocellular bone marrow failure syndromes. Haematologica. 2021;106(1):64-73.

19. Goyal T, Tu ZJ, Wang Z, Cook JR. Clinical and Pathologic Spectrum of DDX41-Mutated Hematolymphoid Neoplasms. Am J Clin Pathol. 2021;156(5):829-838.

20. Singhal D, Hahn CN, Feurstein S, et al. Targeted gene panels identify a high frequency of pathogenic germline variants in patients diagnosed with a hematological malignancy and at least one other independent cancer. Leukemia. 2021;35(11):3245-3256.

21. Li P, White T, Xie W, et al. AML with germline DDX41 variants is a clinicopathologically distinct entity with an indolent clinical course and favorable outcome. Leukemia. 2021.

22. Kim B, Yun W, Lee ST, et al. Prevalence and clinical implications of germline predisposition gene mutations in patients with acute myeloid leukemia. Sci Rep. 2020;10(1):14297.

23. Choi EJ, Cho YU, Hur EH, et al. Unique ethnic features of DDX41 mutations in patients with idiopathic cytopenia of undetermined significance, myelodysplastic syndrome, or acute myeloid leukemia. Haematologica. 2021.

24. Bannon SA, Routbort MJ, Montalban-Bravo G, et al. Next-Generation Sequencing of DDX41 in Myeloid Neoplasms Leads to Increased Detection of Germline Alterations. Front Oncol. 2020;10:582213.

25. Feurstein S, Churpek JE, Walsh T, et al. Germline variants drive myelodysplastic syndrome in young adults. Leukemia. 2021;35(8):2439-2444.

26. Douglas SPM, Siipola P, Kovanen PE, et al. ERCC6L2 defines a novel entity within inherited acute myeloid leukemia. Blood. 2019;133(25):2724-2728.

27. Dirse V, Norvilas R, Gineikiene E, Matuzevičienė R, Griskevicius L, Preiksaitiene E. ETV6 and NOTCH1 germline variants in adult acute leukemia. Leuk Lymphoma. 2018;59(4):1022-1024.

28. Zhang MY, Churpek JE, Keel SB, et al. Germline ETV6 mutations in familial thrombocytopenia and hematologic malignancy. Nat Genet. 2015;47(2):180-185.

29. Nishii R. Molecular basis of ETV6-mediated predisposition to childhood acute lymphoblastic leukemia. Blood. 2020;131(22).

30. Wu W, Liu Y, Zhou Q, et al. Novel homozygous FANCL mutation and somatic heterozygous SETBP1 mutation in a Chinese girl with Fanconi Anemia. Eur J Med Genet. 2017;60(7):369-373.

31. West RR, Calvo KR, Embree LJ, et al. ASXL1 and STAG2 are Common Mutations in GATA2 Deficiency Patients with Bone Marrow Disease and Myelodysplastic Syndrome. Blood Adv. 2021.

32. Kozyra EJ, Göhring G, Hickstein DD, et al. Association of unbalanced translocation der(1;7) with germline GATA2 mutations. Blood. 2021;138(23):2441-2445.

33. Nakazawa H, Yamaguchi T, Sakai H, et al. A novel germline GATA2 frameshift mutation with a premature stop codon in a family with congenital sensory hearing loss and myelodysplastic syndrome. Int J Hematol. 2021;114(2):286-291.

34. Shiba N, Funato M, Ohki K, et al. Mutations of the GATA2 and CEBPA genes in paediatric acute myeloid leukaemia. Br J Haematol. 2014;164(1):142-145.

35. McReynolds LJ, Zhang Y, Yang Y, et al. Rapid progression to AML in a patient with germline GATA2 mutation and acquired NRAS Q61K mutation. Leuk Res Rep. 2019;12:100176.

36. McReynolds LJ, Yang Y, Yuen Wong H, et al. MDS-associated mutations in germline GATA2 mutated patients with hematologic manifestations. Leuk Res. 2019;76:70-75.

37. Fujiwara T, Fukuhara N, Funayama R, et al. Identification of acquired mutations by whole-genome sequencing in GATA-2 deficiency evolving into myelodysplasia and acute leukemia. Ann Hematol. 2014;93(9):1515-1522.

38. Bödör C, Renneville A, Smith M, et al. Germ-line GATA2 p.THR354MET mutation in familial myelodysplastic syndrome with acquired monosomy 7 and ASXL1 mutation demonstrating rapid onset and poor survival. Haematologica. 2012;97(6):890-894.

39. Wang X, Muramatsu H, Okuno Y, et al. GATA2 and secondary mutations in familial myelodysplastic syndromes and pediatric myeloid malignancies. Haematologica. 2015;100(10):e398-401.

40. Fisher KE, Hsu AP, Williams CL, et al. Somatic mutations in children with GATA2-associated myelodysplastic syndrome who lack other features of GATA2 deficiency. Blood Adv. 2017;1(7):443-448.

41. Pastor V, Hirabayashi S, Karow A, et al. Mutational landscape in children with myelodysplastic syndromes is distinct from adults: specific somatic drivers and novel germline variants. Leukemia. 2017;31(3):759-762.

42. Churpek JE, Pyrtel K, Kanchi KL, et al. Genomic analysis of germ line and somatic variants in familial myelodysplasia/acute myeloid leukemia. Blood. 2015;126(22):2484-2490.

43. Ding LW, Ikezoe T, Tan KT, et al. Mutational profiling of a MonoMAC syndrome family with GATA2 deficiency. Leukemia. 2017;31(1):244-245.

44. Galera P, Hsu AP, Wang W, et al. Donor-derived MDS/AML in families with germline GATA2 mutation. Blood. 2018;132(18):1994-1998.

45. Simon L, Spinella JF, Yao CY, et al. High frequency of germline RUNX1 mutations in patients with RUNX1-mutated AML. Blood. 2020;135(21):1882-1886.

46. Ng IK, Lee J, Ng C, et al. Preleukemic and second-hit mutational events in an acute myeloid leukemia patient with a novel germline. Biomark Res. 2018;6:16.

47. Yoshimi A, Toya T, Kawazu M, et al. Recurrent CDC25C mutations drive malignant transformation in FPD/AML. Nat Commun. 2014;5:4770.

48. Brown AL, Arts P, Carmichael CL, et al. RUNX1-mutated families show phenotype heterogeneity and a somatic mutation profile unique to germline predisposed AML. Blood Adv. 2020;4(6):1131-1144.

49. DiFilippo EC, Coltro G, Carr RM, et al. Spectrum of abnormalities and clonal transformation in germline RUNX1 familial platelet disorder and a genomic comparative analysis with somatic RUNX1 mutations in MDS/MPN overlap neoplasms. Leukemia. 2020;34(9):2519-2524.

50. Fournier E, Debord C, Soenen V, et al. Baseline dysmegakaryopoiesis in inherited thrombocytopenia/platelet disorder with predisposition to haematological malignancies. Br J Haematol. 2020;189(4):e119-e122.

51. Duployez N, Martin JE, Khalife-Hachem S, et al. Germline RUNX1 Intragenic Deletion: Implications for Accurate Diagnosis of FPD/AML. Hemasphere. 2019;3(3):e203.

52. Tawana K, Wang J, Király PA, et al. Recurrent somatic JAK-STAT pathway variants within a RUNX1-mutated pedigree. Eur J Hum Genet. 2017;25(8):1020-1024.

53. Kanagal-Shamanna R, Loghavi S, DiNardo CD, et al. Bone marrow pathologic abnormalities in familial platelet disorder with propensity for myeloid malignancy and germline RUNX1 mutation. Haematologica. 2017;102(10):1661-1670.

54. Manchev VT, Bouzid H, Antony-Debré I, et al. Acquired TET2 mutation in one patient with familial platelet disorder with predisposition to AML led to the development of pre-leukaemic clone resulting in T2-ALL and AML-M0. J Cell Mol Med. 2017;21(6):1237-1242.

55. Antony-Debré I, Duployez N, Bucci M, et al. Somatic mutations associated with leukemic progression of familial platelet disorder with predisposition to acute myeloid leukemia. Leukemia. 2016;30(4):999-1002.

56. Sakurai M, Kasahara H, Yoshida K, et al. Genetic basis of myeloid transformation in familial platelet disorder/acute myeloid leukemia patients with haploinsufficient RUNX1 allele. Blood Cancer J. 2016;6:e392.

57. Haslam K, Langabeer SE, Hayat A, Conneally E, Vandenberghe E. Targeted next-generation sequencing of familial platelet disorder with predisposition to acute myeloid leukaemia. Br J Haematol. 2016;175(1):161-163.

58. Staňo Kozubík K, Radová L, Pešová M, et al. C-terminal RUNX1 mutation in familial platelet disorder with predisposition to myeloid malignancies. Int J Hematol. 2018;108(6):652-657.

59. Duarte BKL, Yamaguti-Hayakawa GG, Medina SS, et al. Longitudinal sequencing of RUNX1 familial platelet disorder: new insights into genetic mechanisms of transformation to myeloid malignancies. Br J Haematol. 2019;186(5):724-734.

60. Preudhomme C, Renneville A, Bourdon V, et al. High frequency of RUNX1 biallelic alteration in acute myeloid leukemia secondary to familial platelet disorder. Blood. 2009;113(22):5583-5587.

61. Ernst MPT, Kavelaars FG, Löwenberg B, Valk PJM, Raaijmakers MHGP. RUNX1 germline variants in RUNX1-mutant AML: how frequent? Blood. 2021;137(10):1428-1431.

62. Lachowiez C, Bannon S, Loghavi S, et al. Clonal evolution and treatment outcomes in hematopoietic neoplasms arising in patients with germline RUNX1 mutations. Am J Hematol. 2020;95(11):E313-E315.

63. Mourad S, Bilodeau M, Roussy M, et al. IDH1 as a Cooperating Mutation in AML Arising in the Context of Shwachman-Diamond Syndrome. Front Oncol. 2019;9:772.

64. Lindsley RC, Saber W, Mar BG, et al. Prognostic Mutations in Myelodysplastic Syndrome after Stem-Cell Transplantation. N Engl J Med. 2017;376(6):536-547.

65. Kennedy AL, Myers KC, Bowman J, et al. Distinct genetic pathways define pre-malignant versus compensatory clonal hematopoiesis in Shwachman-Diamond syndrome. Nat Commun. 2021;12(1):1334.

66. Schratz KE, Haley L, Danoff SK, et al. Cancer spectrum and outcomes in the Mendelian short telomere syndromes. Blood. 2020;135(22):1946-1956.
